# Understanding the Care Home Psychiatric Service in North Kent

**DOI:** 10.1192/bjo.2025.10519

**Published:** 2025-06-20

**Authors:** Emily Pettifor, Mohan Bhat

**Affiliations:** Kent and Medway NHS & Social Care Partnership Trust, Dartford, United Kingdom

## Abstract

**Aims:** Over 300,000 people in England and Wales reside in care home settings, with a large proportion of these people thought to have memory difficulties. Within North Kent (Dartford, Gravesham and Swanley) there are 31 care homes, with around 1,540 residents over 65 years old. The North Kent community psychiatric service therefore aims to meet their clinical needs through an in-built care home service, comprised of medical, nursing and support staff. The service facilitates medical reviews and memory assessments, liaises with social care and psychological services, offers care home-based training for staff and supports family carers. The aim of this review was to better understand the patients being supported by this service and interventions being utilised.

**Methods:** A review was completed of the service caseload, documenting patients’ demographics, diagnoses and current medications as of November 2024. Clinical notes were also reviewed to better understand when and why patients had been referred, and what prior contact they may have had with services.

**Results:** Fifty-two caseload patients were identified (around 3% of North Kent older adults in care homes), residing across 20 care homes. All were aged above 60, with the majority in their 80s (44%). Referral was mainly from GP practices (73%), most frequently for support with behavioural or psychological symptoms of Dementia. This can include verbal or physical aggression, agitation, psychotic symptoms and mood disturbance. Referrals were also received for memory assessments, medication advice and support with functional symptoms. Most patients (81%) had never been under the service previously. At the point of caseload review, the majority of patients had formal diagnoses of Dementia (81%). Ongoing intervention was predominantly for medication adjustment and response monitoring (56%). Patients were also receiving behavioural and psychology interventions, and support with depot administration.

Across the caseload, 52% of patients were on antidepressants or mood stabilisers, 42% on benzodiazepines or promethazine and 38% on antipsychotics. NICE guidance advises that for people with Dementia, antipsychotics should only be used if they are severely distressed and at risk of harming themselves or others. We therefore also analysed this for patients with diagnoses of Dementia, of which 33% were on antipsychotics, including risperidone, olanzapine and quetiapine.

**Conclusion:** Through identifying where patients reside, why they were referred and their ongoing needs, we can better understand the population accessing this service, develop links with other community health services and adapt training offered to care home staff to improve the care received by their residents.

